# Supplementary material for: Adaptation in somatosensory afferents improves rate and temporal coding of vibrotactile stimulus features
Source: Commun Biol. 2026 Jul 15;9:1481. doi: 10.1038/s42003-026-10653-7 (PMC13740232; doi:10.1038/s42003-026-10653-7)
Supplement: Supplementary file 1 [file 42003_2026_10653_MOESM1_ESM.pdf]

nature portfolio

# Peer Review File

# Adaptation in somatosensory afferents improves rate and temporal coding of vibrotactile stimulus features

Corresponding Author: Professor Steven Prescott

**This file contains all reviewer reports in order by version, followed by all author rebuttals in order by version.**

Version 0:

Reviewer comments:

Reviewer #1

(Remarks to the Author)
This manuscript investigates the effects of adaptation on neural coding in somatosensory LTMRs, using recordings from rat DRG in response to sinusoidal hind paw stimulation. The authors make two primary claims: (1) that adaptation enhances the coding of stimulus intensity, and (2) that it improves frequency coding via increased phase-locking precision. These findings are potentially interesting—particularly the observations on phase-locking—but some results are not fully convincing in their current form, and parts of the manuscript are difficult to follow.
Major Comments:
• Sample Size and Scope: Please clarify the number of animals and neurons recorded early in the Results section. This information is essential for assessing the robustness of the findings.
• Data Presentation: Some aspects of the Results are challenging to follow. To improve clarity and help readers better grasp the data, I suggest presenting the data from Fig. 1A and Fig. 1C as raster plots. Additionally, consider replotting Fig. 1C to more directly reflect the description in the main text.
• Decoding Analysis: Population vector decoding provides a lower bound and can be substantially inaccurate (e.g., Sanger, 1996). This is particularly relevant for the frequency coding results, where the adaptation effect appears small/absent. A complementary analysis at the single-cell level could help address this concern, as information sampling is less of an issue and more accurate methods are available (e.g., Panzeri et al., 2007).
• Fig. 2 Analysis: It appears that the analysis in Fig. 2 is based on a single sample of 8 neurons. If this is incorrect, please clarify. If accurate, this sample size may be insufficient to support a general conclusion.
• Line 121 and Following: The hypothesis is not clearly articulated.
• Phase-Locking Effects: The most compelling aspect of the paper is the effect of adaptation on phase-locking.Fig. 5B suggests this is a property of some LTMRs but not all LTMRs. Additional analysis—such as quantifying changes in jitter from early to late during the stimulus—could help.
• Contextualization with Prior Work: The manuscript would benefit from a discussion of how these findings relate to prior work on adaptation and coding in the whisker somatosensory system. Relevant studies include those by Stanley (e.g., Wright et al., 2021 and several others), Lampl (reviews are cited), Petersen (Colins Rodriguez et al., 2024), and Maravall (Maravall et al., 2013).
Minor Comments:
• The abstract is somewhat difficult to follow when read in isolation. The summary at the end of the Introduction is clearer and could serve as a model for revising the abstract.
• Please specify how many repetitions of the 30-second stimulus were presented. Including this in the Results section would be helpful.

Reviewer #2

(Remarks to the Author)
In this study, the authors examined how the intensity and frequency of tactile stimulation to the paw are encoded by low-threshold mechanoreceptors (LTMRs). They use machine learning methods to assess how these features are encoded in response to vibrotactile stimulation of the hind limb. They show that: 1) The firing rate of these neurons drops due to adaptation, and this increases the non-uniformity of the response across the population. 2) Rate coding of stimulus intensity rapidly improves due to adaptation and then slowly declines, whereas encoding of stimulus frequency does not improve

much at early time points but is reduced more quickly over longer timescales. 3) At the population level, the improvement in rate coding of intensity is due to non-uniform adaptation. 4) Temporal coding performs better for decoding stimulus frequency compared to intensity, and its performance is barely affected by adaptation. 5) Adaptation leads to fewer spikes per stimulus cycle, but these are better-timed spikes, improving temporal coding and increasing the informativeness of individual spikes. 6) Frequency coding is strongly affected by jitter, and adaptation specifically improves the coding of stimulus frequency. As a result, coding efficiency improves in terms of information per spike (i.e., metabolic efficiency). 7) Adaptation greatly improves simultaneous coding of both intensity and frequency. Moreover, temporal coding is superior to rate coding in this simultaneous coding of intensity and frequency.
Since all of these findings were discovered in first-order cells, this implies that neither synaptic nor network mechanisms are required for the generation of these coding schemes. This is a great paper that addresses classical and fundamental questions in the field of sensory coding, namely rate vs. temporal coding and how both are involved in coding of two major features of tactile stimulation: intensity and frequency. The methods are clear, the experimental design is excellent, and the findings are well described and entirely convincing.
Despite these strong achievements, there are several limitations that should be considered. The work is limited to anesthetized animals. It remains unclear how downstream neurons utilize the coding schemes demonstrated in first-order cells. The underlying mechanisms of adaptation were not directly investigated, and the behavioral relevance of the findings was not explored. Nonetheless, this is a very strong study, and I fully support the publication of this paper. Below, I list a few comments that may be viewed as suggestions for further improvement.
Comments and Suggestions:
1) Although the authors provide references for the recording methods from earlier papers, they may wish also to summarize this information directly in the current manuscript.
2) The study does not examine whether individual LTMRs preferentially encode stimulus features using rate versus temporal codes, or whether they are more tuned to stimulus intensity versus frequency. Furthermore, it remains unclear whether the ability of classifiers to decode the stimulus depends on specific and limited number of neurons—some of which may contribute strongly, while others contribute little. Identifying such specialization, if possible, could reveal important functional diversity within the afferent population, and the authors may wish to consider collecting additional recordings, if needed, to address this question more systematically.
3) As a suggestion: since multielectrode arrays were used, the authors could ask whether coordinated activity across simultaneously recorded neurons contributes in any way to stimulus coding.
4) Another useful analysis would be to assess how well stimulus features can be decoded on a single-trial basis, and how this performance scales with the number of neurons included. The authors could pool responses from varying-sized subsets of neurons and assess decoding accuracy across different random combinations, even within their limited dataset. This would provide insight into the efficiency, robustness, and potential redundancy of the population code. It would also clarify whether adaptation enhances coding at the level of individual trials, which more closely reflects the demands of real-time sensory processing.

Version 1:

Reviewer comments:

Reviewer #1

(Remarks to the Author)
The authors have addressed my previous comments in a thorough manner and the manuscript is markedly improved. I have a few residual minor points:

Although improved, parts of the Abstract are likely to be hard to understand for readers coming fresh to the work. “Rate coding of stimulus intensity was improved via non-uniform (i.e. stimulus-dependent) changes in tuning; this improvement was absent if firing rate was reduced in an artificially uniform manner”. In the context of the abstract, this is not clear what is being . Also, the final sentence would be more useful if it spells out what the “different mechanisms” are.

91. “Purple rasters in Figure 1A illustrate this: responses to 100 Hz stimulation strongly adapt whereas responses to 50 Hz stimulation do not”. This is confusing, since the text leads the read to expect to see adaptation but the cartoon responses shown appear stationary in time (no decrease in spiking with time is evident).

Fig 2b. UMAP embeddings capture local structure in the source space but only partially show global structure . Hence, this does not add much evidence for separability.

Fig3a. real/simulated labels - I was confused by this. Are the labels the wrong way round?

Reviewer #2

(Remarks to the Author)
The authors have adequately addressed my major concerns, and I therefore recommend acceptance of the manuscript in its current form. This is a rigorous and well-executed study with important implications for understanding how the somatosensory system encodes vibrotactile stimuli.

**Open Access** This Peer Review File is licensed under a Creative Commons Attribution 4.0 International License, which permits use, sharing, adaptation, distribution and reproduction in any medium or format, as long as you give appropriate credit to the original author(s) and the source, provide a link to the Creative Commons license, and indicate if changes were made.
In cases where reviewers are anonymous, credit should be given to 'Anonymous Referee' and the source.
The images or other third party material in this Peer Review File are included in the article's Creative Commons license, unless indicated otherwise in a credit line to the material. If material is not included in the article's Creative Commons license and your intended use is not permitted by statutory regulation or exceeds the permitted use, you will need to obtain permission directly from the copyright holder.
To view a copy of this license, visit https://creativecommons.org/licenses/by/4.0/

We thank the reviewers for their constructive feedback. Their comments are reproduced verbatim below, in black. Comments have been numbered for clarity when cross-referencing our responses to them. Our responses are provided in blue. All changes to the manuscript are tracked in red. We have striven to address all the reviewer concerns. Beyond expanding and refining our text, we have also added new analysis to Figures 2, 4, and 5. We also enhanced Figure 1 to help introduce key concepts. We believe the revised paper is much improved, and we hope it will be deemed suitable for publication.

**Reviewer #1 (Remarks to the Author):**

This manuscript investigates the effects of adaptation on neural coding in somatosensory LTMRs, using recordings from rat DRG in response to sinusoidal hind paw stimulation. The authors make two primary claims: (1) that adaptation enhances the coding of stimulus intensity, and (2) that it improves frequency coding via increased phase-locking precision. These findings are potentially interesting—particularly the observations on phase-locking—but some results are not fully convincing in their current form, and parts of the manuscript are difficult to follow.

**Major Comments:**

1.1 - Sample Size and Scope: Please clarify the number of animals and neurons recorded early in the Results section. This information is essential for assessing the robustness of the findings.

We added the number of rats and recorded units in the Methods (lines 378-392). The Methods seemed like the best location to explain why the number of units used for studying rate- and ISI-based codes differ. Nevertheless, to ensure readers see this information when first reading the Results, we also added the numbers to the figure legends (lines 776-777 and 813-814). Relevant information was also added to the Results (line 147) when explaining newly added analysis in Figures 2D,E.

1.2 - Data Presentation: Some aspects of the Results are challenging to follow. To improve clarity and help readers better grasp the data, I suggest presenting the data from Fig. 1A and Fig. 1C as raster plots. Additionally, consider replotting Fig. 1C to more directly reflect the description in the main text.

We added rasters to Figure 1 as panel A. This panel was designed to introduce concepts essential for understanding rate- and ISI-based coding, including how ambiguous representations can arise and how adaptation might resolve that ambiguity. For clarity, we used cartoon rasters instead of experimentally recorded data. The new text (lines 73-93) was designed to ease readers into the other graphs presented Figure 1. Graphs in panels B and C illustrate how firing rate changes over time due to adaptation, including how those changes depend on stimulus frequency (panel B) or intensity (panel C). Illustrating how effects of adaptation depend on stimulus parameters is important for explaining "non-uniform" effects in panel D (and, notably, is now illustrated in panel A). Such changes are far less evident on experimental rasters, which include 1000s of spikes if plotted for the entire 30 s response. We also think tuning curves in panel D should be clearer now that entrainment is better explained in panel A. Tuning curves and non-uniform effects of adaptation appear again in Fig 3, and so introducing them in Fig 1 is important.

In the original figure, we organized panel C (now D) so that neurons (#3, 46, 47, and 18) are depicted in the same order they are mentioned in the text. This panel illustrates how data shown in earlier panels can be re-plotted as tuning curves, which includes converting firing rate to entrainment, effectively normalizing firing rate by stimulus frequency and thus clarifying the effect of stimulus intensity on evoked spiking. By showing tuning curves before and after adaptation, the reader can see that effects of adaptation differ across stimulus intensities and frequencies, and across cells, foreshadowing Fig 3.

Some of our changes may not match exactly what the reviewer had in mind, but we think they address

the reviewer's core concerns about helping readers appreciate the nature of our data, including how to conceptualize different coding schemes and the potential benefits of adaptation.

1.3 - Decoding Analysis: Population vector decoding provides a lower bound and can be substantially inaccurate (e.g., Sanger, 1996). This is particularly relevant for the frequency coding results, where the adaptation effect appears small/absent. A complementary analysis at the single-cell level could help address this concern, as information sampling is less of an issue and more accurate methods are available (e.g., Panzeri et al., 2007).

We did not repeat trials of the same stimulus (i.e. with the same intensity and frequency) in a given neuron. Consequently, we cannot estimate noise entropy. Based on separate tests in LTMRs, we know that their firing rate is quite consistent across repeated trials (much more so than in cortical neurons). This is similarly true for the ISI distribution despite trial-to-trial variations in the precise spike pattern (i.e. the ISI sequence differs because of quasi-random skipping, but the skipping is statistically consistent). Accordingly, noise entropy is low for both our rate- and ISI-based measures. Nevertheless, we acknowledge that not accounting for noise entropy (including variations across different LTMRs) affects mutual information in absolute terms, but the impact for our study is mitigated by our focus on differences in mutual information due to adaptation; in other words, the errors likely cancel out. Long story short, we cannot apply the bias correction methods outlined by Panzeri et al. (2007).

To be transparent about these issues, we now explain in the Methods (lines 372-378) (i) that multiple trials of the same stimulus were not repeated, (ii) that this precludes us from measuring noise entropy, and (iii) that this impacts the accuracy of our mutual information values. We also explain that this should not severely impact our interpretation, which relies on changes in information due to adaptation. We also explain our decision not to repeat trials, because our recordings were of limited duration – bearing in mind that each stimulus trial was long (to induce adaptation) and inter-trial intervals were long (to allow recovery from adaptation) – which means repeating the same stimulus would have necessarily entailed testing fewer different stimulus intensity-frequency combinations.

The reviewer's comment focussed on corrections for sampling bias, but Panzeri et al. (2007) also addressed shuffling. As now explained (lines 393-404), we did not record simultaneously from >1 neuron, and so our data are not subject to noise correlations and, therefore, shuffling is unnecessary.

1.4 - Fig. 2 Analysis: It appears that the analysis in Fig. 2 is based on a single sample of 8 neurons. If this is incorrect, please clarify. If accurate, this sample size may be insufficient to support a general conclusion.

Yes, the analysis in Figure 2 is based on data from the 8 units for which data was collected for all stimulus amplitude-frequency combinations (see response to point 1.1). We have now expanded analysis to include data from all 26 recorded units, and have added this to Figure 2 as panels D and E. The new graphs, which show decoding accuracy for different ensemble sizes, confirms that our original results generalize to different ensemble sizes. Please also see responses below to points 2.2 and 2.5.

1.5 - Line 121 and Following: The hypothesis is not clearly articulated.

To clarify the hypothesis (now on lines 156-158), we refined the associated text (lines 158-166). Beyond illustrating what we mean by uniform and non-uniform adaptation in Figure 3A, we designed the panel added to Fig 1 (see point 1.2) with this hypothesis in mind; specifically, in the cartoon, the response to 50 Hz stimulation does not adapt (compare black and purple rasters) whereas the response to 100 Hz stimulation does. This non-uniform (i.e. stimulus-dependent) adaptation leads to evoked firing rates that are unique for each stimulus intensity-frequency combination, thus improving decodability. The later

text (line 166) was modified to clarify our prediction – that artificially uniform adaptation will NOT improve decoding, unlike realistically non-uniform adaptation. Figure 3B confirms that prediction. Collectively, these changes should significantly improve the clarity of our presentation.

1.6 - Phase-Locking Effects: The most compelling aspect of the paper is the effect of adaptation on phase-locking.Fig. 5B suggests this is a property of some LTMRs but not all LTMRs. Additional analysis—such as quantifying changes in jitter from early to late during the stimulus—could help.

We regret if we have misunderstood the reviewer's comment, but Fig 5B reports changes in jitter during the 30-s-long stimulus. Absolute jitter (in milliseconds) decreases as stimulus frequency increases, but jitter relative to the stimulus period tends to remain constant, hence normalizing jitter by stimulus period (i.e. reporting it as phase) helps remove the effects of stimulus frequency, thus enabling data for different stimulus frequencies to be pooled to better highlight effects of adaptation in each cell. And so, this is precisely how we plotted data in Fig 5B. The reviewer is correct that not all cells exhibit the same change in jitter over time, but the inset shows that the average reduction in jitter is sizeable. We also tested different artificially introduced jitters in Fig S6 and reproduced the effect for a wide range of neural ensemble sizes (Fig 5E).

1.7 - Contextualization with Prior Work: The manuscript would benefit from a discussion of how these findings relate to prior work on adaptation and coding in the whisker somatosensory system. Relevant studies include those by Stanley (e.g., Wright et al., 2021 and several others), Lampl (reviews are cited), Petersen (Colins Rodriguez et al., 2024), and Maravall (Maravall et al., 2013).

As suggested, more detailed consideration of whisker somatosensory processing has been integrated into the Discussion as a new paragraph (lines 286-297).

**Minor Comments:**

1.8 - The abstract is somewhat difficult to follow when read in isolation. The summary at the end of the Introduction is clearer and could serve as a model for revising the abstract.

We re-wrote the Abstract as suggested by the reviewer (lines 21-32). We also reduced the word count to fit within the 150-word maximum.

1.9 - Please specify how many repetitions of the 30-second stimulus were presented. Including this in the Results section would be helpful.

As now explained under point 1.3, trials of the same stimulus intensity-frequency combination were not repeated. This is now explained in the revised Methods (lines 372-378).

**Reviewer #2 (Remarks to the Author):**

In this study, the authors examined how the intensity and frequency of tactile stimulation to the paw are encoded by low-threshold mechanoreceptors (LTMRs). They use machine learning methods to assess how these features are encoded in response to vibrotactile stimulation of the hind limb. They show that: 1) The firing rate of these neurons drops due to adaptation, and this increases the non-uniformity of the response across the population. 2) Rate coding of stimulus intensity rapidly improves due to adaptation and then slowly declines, whereas encoding of stimulus frequency does not improve much at early time points but is reduced more quickly over longer timescales. 3) At the population level, the improvement in rate coding of intensity is due to non-uniform adaptation. 4) Temporal

coding performs better for decoding stimulus frequency compared to intensity, and its performance is barely affected by adaptation. 5) Adaptation leads to fewer spikes per stimulus cycle, but these are better-timed spikes, improving temporal coding and increasing the informativeness of individual spikes. 6) Frequency coding is strongly affected by jitter, and adaptation specifically improves the coding of stimulus frequency. As a result, coding efficiency improves in terms of information per spike (i.e., metabolic efficiency). 7) Adaptation greatly improves simultaneous coding of both intensity and frequency. Moreover, temporal coding is superior to rate coding in this simultaneous coding of intensity and frequency.

Since all of these findings were discovered in first-order cells, this implies that neither synaptic nor network mechanisms are required for the generation of these coding schemes. This is a great paper that addresses classical and fundamental questions in the field of sensory coding, namely rate vs. temporal coding and how both are involved in coding of two major features of tactile stimulation: intensity and frequency. The methods are clear, the experimental design is excellent, and the findings are well described and entirely convincing.

Despite these strong achievements, there are several limitations that should be considered. The work is limited to anesthetized animals. It remains unclear how downstream neurons utilize the coding schemes demonstrated in first-order cells. The underlying mechanisms of adaptation were not directly investigated, and the behavioral relevance of the findings was not explored. Nonetheless, this is a very strong study, and I fully support the publication of this paper. Below, I list a few comments that may be viewed as suggestions for further improvement.

We thank the reviewer for this extensive summary. We tried to highlight in our original Discussion the limitations that the reviewer points out, but we have made small changes to point this out more clearly, including for adaptation mechanisms (line 330) and behavioral consequences (lines 282-284). We have also added significant new text to the Discussion to address the issue of ensemble size, how this might be defined by the nature of downstream processing (lines 330-340). This is triggered in large part by new analysis that shows discrimination (and the benefits of adaptation on discrimination) varies with ensemble size (see responses to 2.2 and 2.4).

**Comments and Suggestions:**

2.1 - Although the authors provide references for the recording methods from earlier papers, they may wish also to summarize this information directly in the current manuscript.

We have added significant new detail to the Methods (lines 372-404). The includes the number of units recorded and explains why certain data sets were incomplete, and how this impacted our analysis. The added detail is intended to provide helpful context while remaining concise.

2.2 - The study does not examine whether individual LTMRs preferentially encode stimulus features using rate versus temporal codes, or whether they are more tuned to stimulus intensity versus frequency. Furthermore, it remains unclear whether the ability of classifiers to decode the stimulus depends on specific and limited number of neurons—some of which may contribute strongly, while others contribute little. Identifying such specialization, if possible, could reveal important functional diversity within the afferent population, and the authors may wish to consider collecting additional recordings, if needed, to address this question more systematically.

We recently described rodent LTMR tuning in detail (Medlock et al. J Neurosci 2024). We reported that “unlike the dichotomous frequency preference characteristic of RA1 and RA2/Pacinian afferents in other

species, rodent RAs fell along a continuum". Accordingly, all LTMRs responded across most stimulus frequencies and intensities (for the ranges we tested), thus diminishing concerns about certain neurons contributing disproportionately to decoder performance.

Nevertheless, this concern is dealt with most directly be increasing the sample size. Instead of testing new cells, we expanded certain analysis to include all 26 recorded neurons. As now explained (see response to 1.1, analysis of rate-base coding (in Figures 2 and 3) focused on 8 neurons for which all stimulus intensity-frequency combinations had been tested, but having established our analysis tools under these strict conditions, we adapted the analysis to accommodate some missing data points so data from many more neurons could be included.

We added this new analysis – decoder accuracy as a function of group size – as Fig 2D and E. The results show that adaptation improves decoding of stimulus intensity for small groups of neurons but, as performance approaches near-perfect for larger groups, the benefit was occluded by a ceiling effect. In contrast, adaptation did not improve decoding of stimulus frequency from firing rate for any ensemble size, and often worsened performance. Given that different neurons were subsampled for inclusion in small groups, we can confidently conclude that our results are not skewed by certain neurons having large effects. All of this is now addressed in the revised text (lines 146-154) and figure legends (lines 785-792). The original analysis of ISI-based coding included data from all 26 neurons, but we repeated the same analysis of decoder performance as a function of group size for inclusion in Figs 4E and 5E. See also response to point 2.4

2.3 - As a suggestion: since multielectrode arrays were used, the authors could ask whether coordinated activity across simultaneously recorded neurons contributes in any way to stimulus coding.

Despite using multielectrode arrays, we rarely recorded simultaneously from two or more LTMRs with overlapping receptive fields (which is necessary to co-activate them). Inertia limits the probe size that can be used when stimulating at high frequency. Also, we prioritized "clean" recordings, which means electrode position was adjusted to obtain the best possible recording from one unit rather than obtaining poorer quality recordings from 2 or more units. This was to ensure spike sorting was both sensitive and specific because missing (false negative) or extra (false positive) spikes can severely compromise our analysis, especially the ISI distribution. This is now mentioned in the revised Methods (lines 393-404).

2.4 - Another useful analysis would be to assess how well stimulus features can be decoded on a single-trial basis, and how this performance scales with the number of neurons included. The authors could pool responses from varying-sized subsets of neurons and assess decoding accuracy across different random combinations, even within their limited dataset. This would provide insight into the efficiency, robustness, and potential redundancy of the population code. It would also clarify whether adaptation enhances coding at the level of individual trials, which more closely reflects the demands of real-time sensory processing.

We are wary of trying to quantify decoding on a single-trial basis. As now more clearly explained (see above), we are analyzing responses as if neurons (which are separately recorded) are operating as part of the same ensemble. This precludes us from considering noise correlations, and nor do we have any psychophysical data that might motivate a deeper dive into trial-to-trial variability. That said, we agree there is great value in looking at the effects of ensemble size, but we have focussed on how average performance scales with ensembles size

We added new analysis for rate-based coding (as explained under point 2.2) and we also added new analysis for ISI-based coding. These results are now shown as Figs 2D, 2E, 4E and 5E. When decoding from firing rate, we found that adaptation improves the discrimination of stimulus intensity for all

ensemble sizes (Fig. 2D), although the effect shrinks for large ensembles as performance of non-adapted ensembles becomes nearly perfect, thus introducing a ceiling effect. In contrast, decoding of stimulus frequency from firing rate shows that adaptation is not beneficial for any ensemble size, but instead often degrades performance (Fig. 2E). The results are reported on lines 146-154.

When decoding from ISI distributions, we focussed on decoding stimulus frequency. The results show that adaptation was beneficial for ensembles of >10 neurons but was detrimental for ensembles of <6 neurons. This is reported in Fig. 4E and on lines 202-204 of the Results. The effects of adaptation on the temporal code were nullified when artificially increasing the jitter of spike-times for all ensemble sizes that previously exhibited a benefit (Fig. 5E), as explained on line 238.

**Reviewer #1** (Remarks to the Author):
The authors have addressed my previous comments in a thorough manner and the manuscript is markedly improved. I have a few residual minor points:

Although improved, parts of the Abstract are likely to be hard to understand for readers coming fresh to the work. “Rate coding of stimulus intensity was improved via non-uniform (i.e. stimulus-dependent) changes in tuning; this improvement was absent if firing rate was reduced in an artificially uniform manner”. In the context of the abstract, this is not clear what is being . Also, the final sentence would be more useful if it spells out what the “different mechanisms” are.
The first point about uniform changes is addressed on lines 27-28, where we now instead write: “Rate coding of stimulus intensity was improved when firing rate was differentially modulated depending on stimulus features because this altered tuning non-uniformly across neurons.”

The two mechanisms are now spelled out on line 31, where we now write: tuning curve alterations and jitter reduction.

Slight changes were made elsewhere in the Abstract to keep the word count to 150.

91. “Purple rasters in Figure 1A illustrate this: responses to 100 Hz stimulation strongly adapt whereas responses to 50 Hz stimulation do not”. This is confusing, since the text leads the read to expect to see adaptation but the cartoon responses shown appear stationary in time (no decrease in spiking with time is evident).
In the figure legend (line 759-760), we now write that: slow effects of adaptation are not evident as a gradual change in firing rate over the short (120 ms-long) epochs illustrated [in Figure 1a].

Fig 2b. UMAP embeddings capture local structure in the source space but only partially show global structure. Hence, this does not add much evidence for separability.
We thoroughly quantified separability using decoding accuracy and information. The UMAP embeddings are used only to help readers visualize how adaptation helps improve separability. This is now mentioned in the Methods (line 456). Our description of UMAPs in the Results is consistent with that (e.g. line 782-783: UMAPs illustrate that rate vectors for different stimulus intensities are more linearly separable after adaptation, which supports better decoding).

Fig3a. real/simulated labels - I was confused by this. Are the labels the wrong way round?
We have confirmed that the labels on Fig 3a are correct. The uniform adaptation in simulated data is evident as curves that are all equally “squished down” relative to the initial data shown in the top panel. The non-uniform adaptation in real data is evident from each curve changes slightly differently from the next.

**Reviewer #2** (Remarks to the Author):
The authors have adequately addressed my major concerns, and I therefore recommend acceptance of the manuscript in its current form. This is a rigorous and well-executed study with important implications for understanding how the somatosensory system encodes vibrotactile stimuli.
