# Supplementary material for: Adaptation in somatosensory afferents improves rate and temporal coding of vibrotactile stimulus features
Source: Commun Biol. 2026 Jul 15;9:1481. doi: 10.1038/s42003-026-10653-7 (PMC13740232; doi:10.1038/s42003-026-10653-7)
Supplement: Supplementary file 2 [file 42003_2026_10653_MOESM2_ESM.pdf]

SUPPLEMENTAL FIGURES

Adaptation in somatosensory afferents improves rate and temporal coding of vibrotactile stimulus features

Adel Halawa, Christopher Dedek, Laura Medlock, Dhekra Al-Basha, Stéphanie Ratté, Steven A Prescott

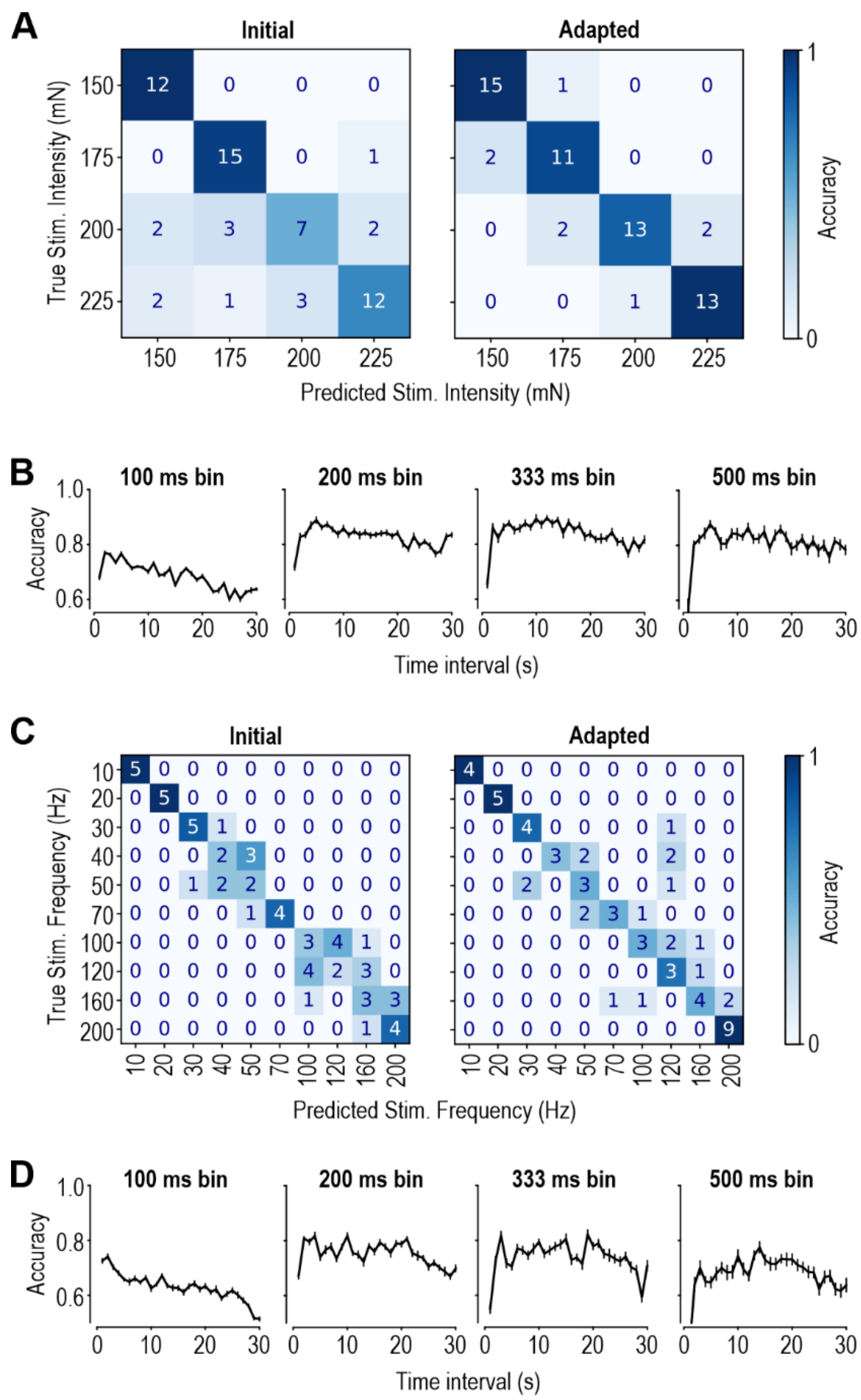

Figure S1. Extended analysis of decoding from population rate vectors.
A. Correlation matrices for actual and decoded stimulus intensity based on data from the first second of stimulation (initial, left) compared to the 30th second (adapted, right). This is one of the 20 test sets generated for Fig. 2B.
B. Accuracy of intensity decoding when spikes are counted in different width bins. The 200 ms integration window is the same data as in Fig. 2B.
C,D. Same as A and B, but for decoding stimulus frequency. Compare to Fig. 2C.

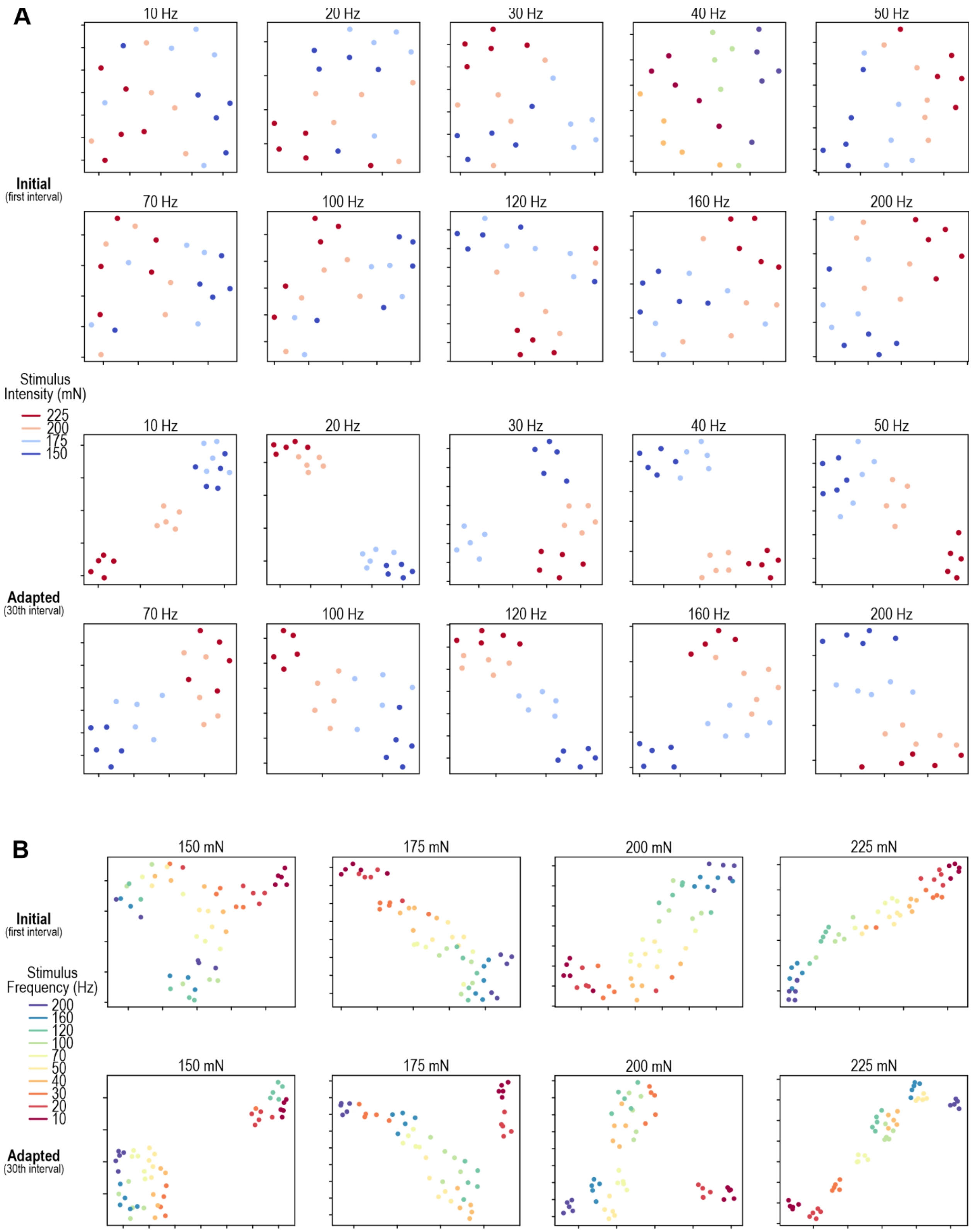

Figure S2. UMAP embeddings of population firing rate vectors before and after adaptation. **A.** UMAP embeddings of rate vectors for different stimulus intensities (color). Each panel shows

rate vectors for a different stimulus frequency. Rate vectors for different stimulus intensities are more linearly separable after adaptation (bottom two rows) than initially (top two rows).

B. Same as A but for rate vectors for different stimulus frequencies (color). Each panel shows rate vectors for a different stimulus intensity. Unlike in A, rate vectors for different stimulus frequencies are not more linearly separable after adaptation (bottom row) than initially (top row).

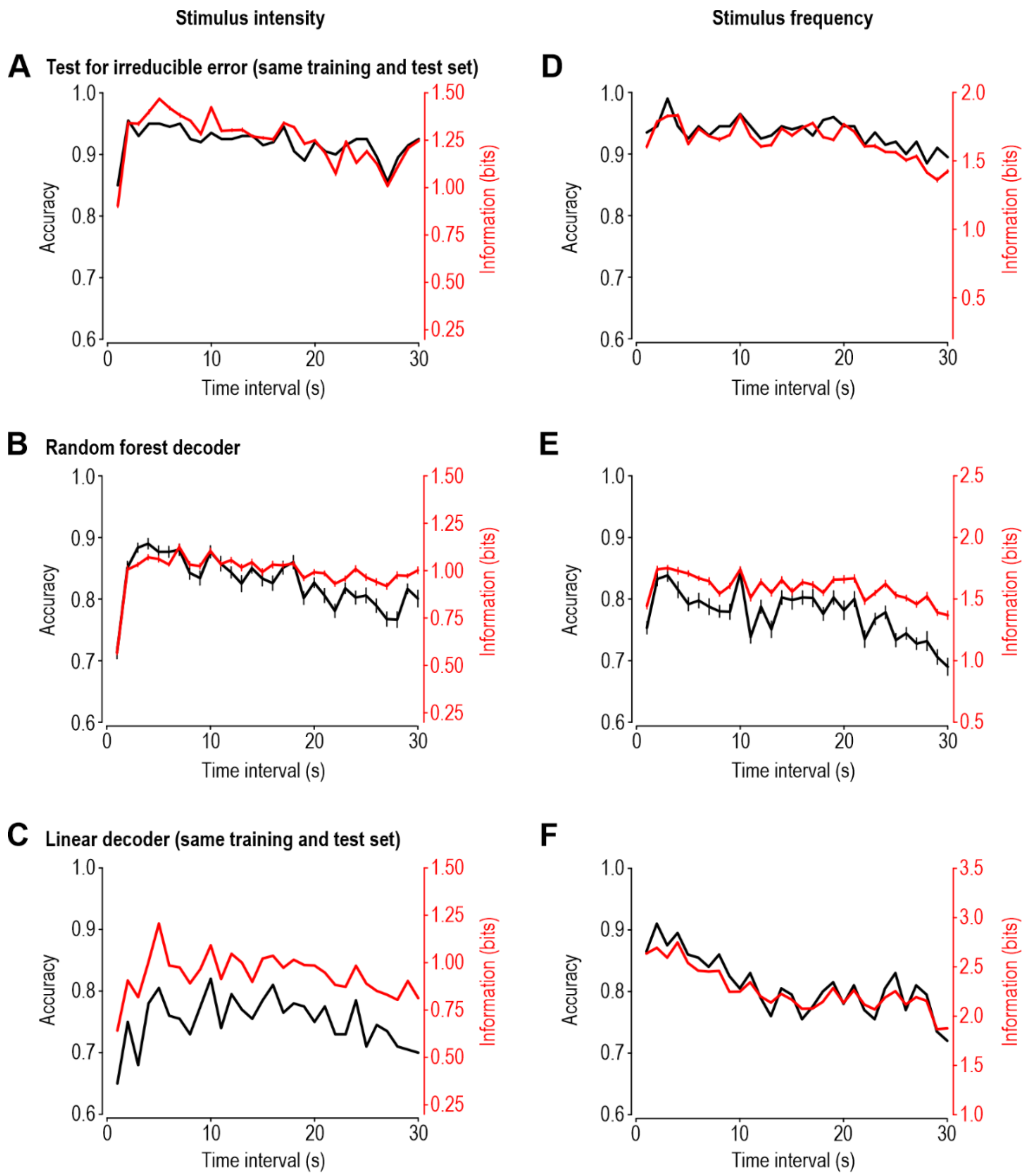

Figure S3. Comparison of decoders to assess discriminability of stimulus intensity (A-C) or frequency (D-F) from population firing rate.

A. Same as Fig. 2B (decoding stimulus intensity) using an SVC but with the training set equal to the test set. This approach removes error due to failure of the model to generalize from the training set to the test set, and thus isolates the contribution of irreducible error (i.e. inability to discriminate different stimuli because they evoke similar firing rates).

B. Same as Fig. 2B but using a random forest decoder instead of an SVC. The train/test split (0.7/0.3) is repeated 20 times with different splits.

C. Same as Fig. 2B but using a linear decoder instead of an SVC. Training set is equal to test set.

D-F. Same as Fig. 2C (decoding stimulus frequency) using an SVC but with the same training and test set (**D**), a random forest decoder (**E**), or a linear decoder (**F**).

Regardless of the decoder, rate-based coding of stimulus intensity benefited from adaptation whereas rate-based coding of stimulus frequency did not.

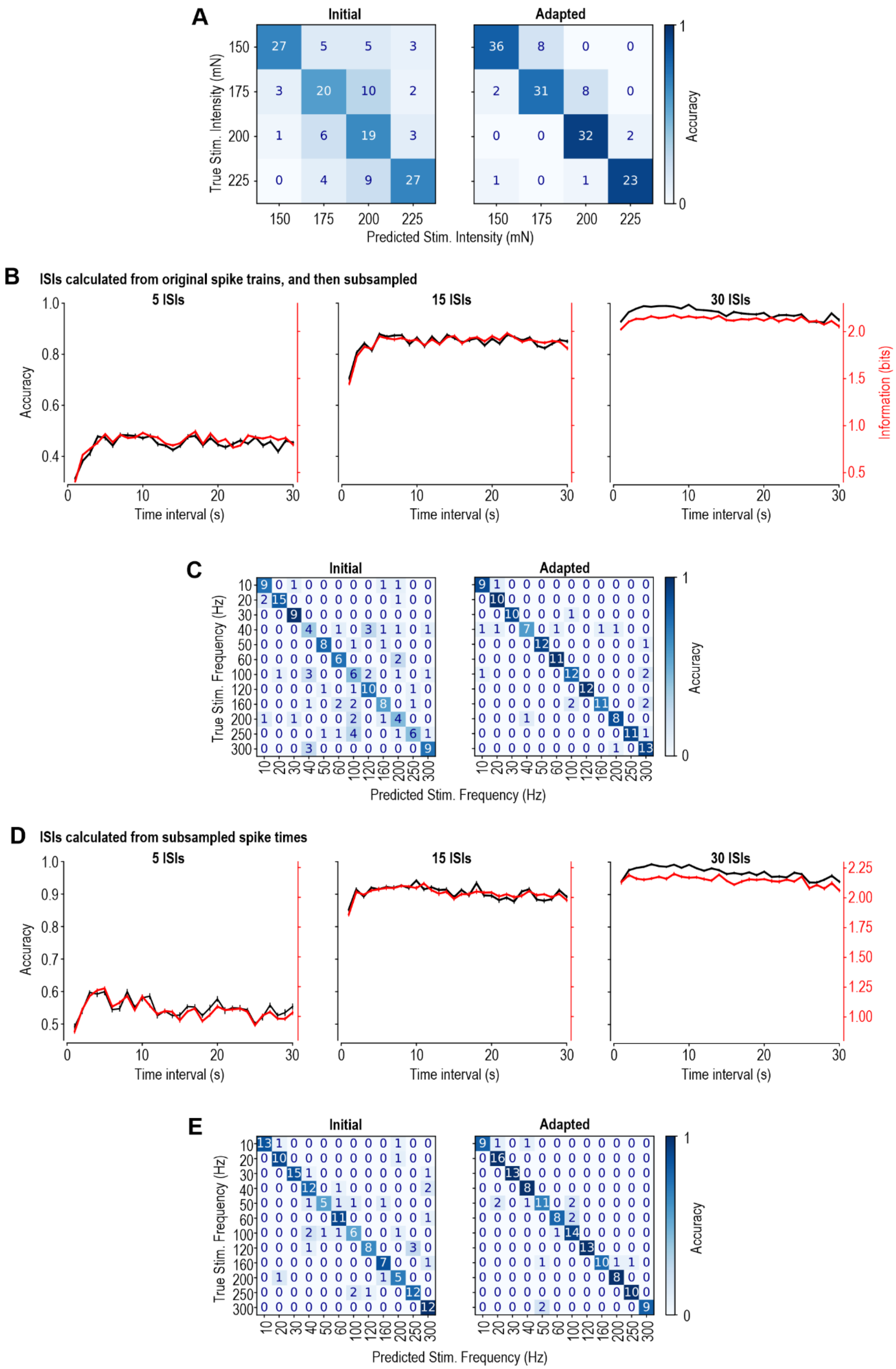
A
Initial
Adapted
True Stim. Intensity (mN)
Predicted Stim. Intensity (mN)
150
175
200
225
27 5 5 3
3 20 10 2
1 6 19 3
0 4 9 27
36 8 0 0
2 31 8 0
0 0 32 2
1 0 1 23
Accuracy
B
ISIs calculated from original spike trains, and then subsampled
5 ISIs
15 ISIs
30 ISIs
Accuracy
Information (bits)
Time interval (s)
C
Initial
Adapted
True Stim. Frequency (Hz)
Predicted Stim. Frequency (Hz)
Accuracy
D
ISIs calculated from subsampled spike times
5 ISIs
15 ISIs
30 ISIs
Accuracy
Time interval (s)
E
Initial
Adapted
True Stim. Frequency (Hz)
Predicted Stim. Frequency (Hz)
Accuracy

Figure S4. Extended analysis of decoding from ISI distributions.
A. Correlation matrices for actual and decoded stimulus intensity based on data from the first second of stimulation (left) compared to the 30th second (right) for one of the 20 test sets used to generate Fig. 4C.
B. Accuracy of frequency decoding when using different numbers of subsampled ISIs (5, 15, or 30 ISIs from left to right). Pale data in Fig. 4D corresponds to data for 15 ISIs shown here.
C. Correlation matrices for actual and decoded stimulus frequency based on data from the first second of stimulation (left) compared to the 30th second (right) from one of the test sets using 15 subsampled ISIs, like in panel B
D. Same as B, but instead of subsampling ISIs after calculating them, spikes times were subsampled to yield the target number of ISIs (5, 15, or 30), and ISIs were then calculated from subsampled spike times (see Methods). The same improvement in decoding is seen.
E. Same as C, but with the data from the middle panel of part D, when the subsample number is equal to 15 ISIs.

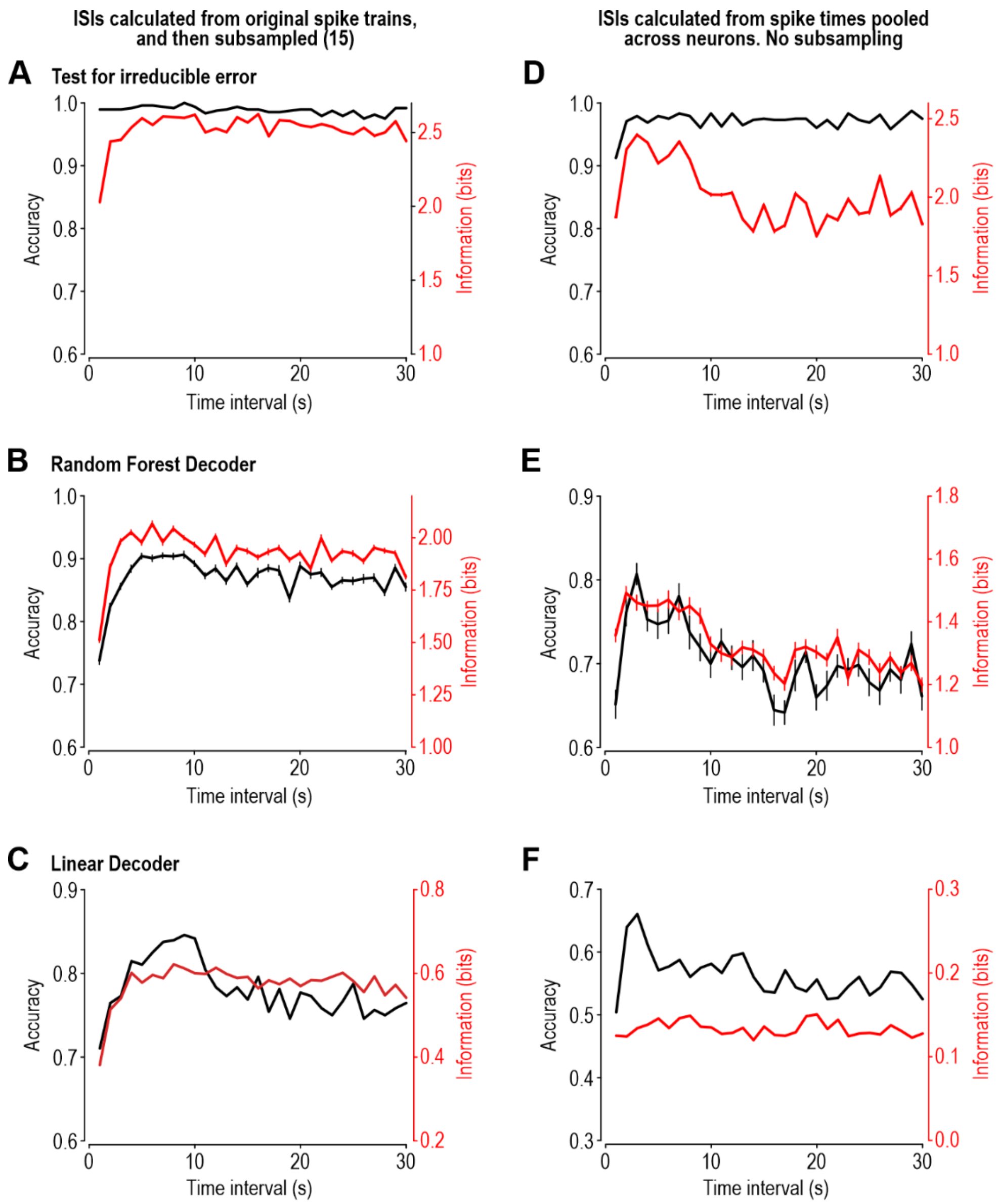

Figure S5. Comparison of decoders to assess discriminability of stimulus frequency from 15 ISIs subsampled from ISIs calculated from original spike trains (A-C) or from all ISIs calculated after spike times were pooled across neurons (D-F).

A. Same as Fig. 4D pale but using an SVC but with the training set equal to the test set.

B. Same as Fig. 4D pale but using a random forest decoder instead of an SVC. The train/test split (0.7/0.3) was repeated 20 times with different splits.

C. Same as Fig. 4D pale but using a linear decoder instead of an SVC. The train/test split (0.7/0.3) was repeated 20 times with different splits.

D-F. Same as A-C, respectively, but using ISIs calculated after spike times were pooled across neurons, like in Figure 4F.

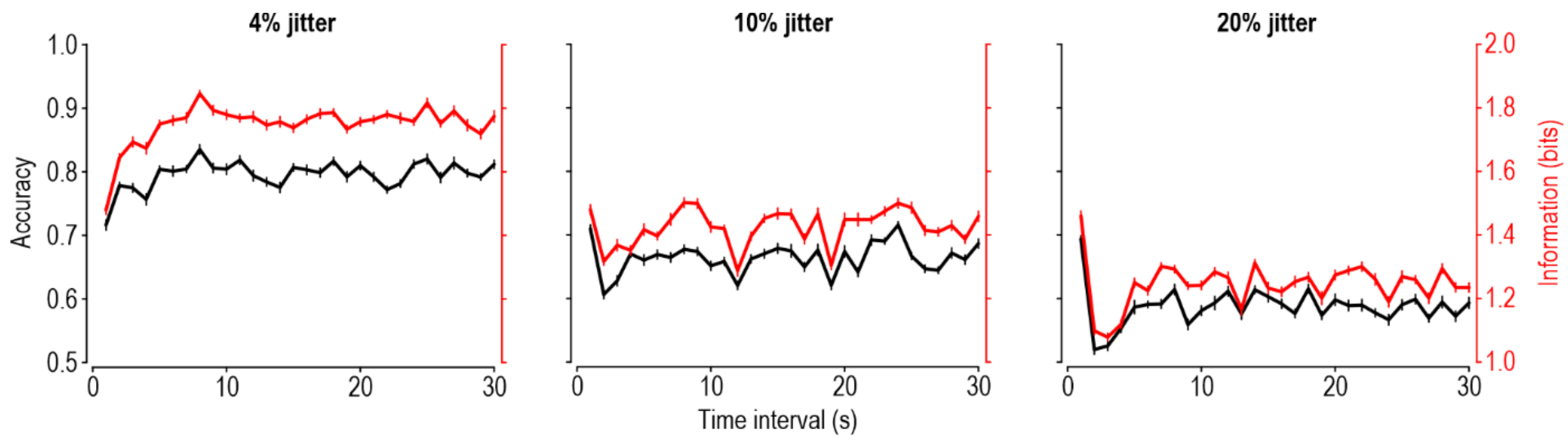

Figure S6. Extended analysis of effect of artificially altering phase-locking precision. Same as Fig. 5C pale (decoding of stimulus frequency from ISI distribution after jittering), but for spike times jittered by different amounts. Jitter spike times by <6% (left) did not diminish the gains in decodability afforded by adaptation, while strong jitter did (center and right).
