# Supplementary material for: Adaptation in somatosensory afferents improves rate and temporal coding of vibrotactile stimulus features
Source: Commun Biol. 2026 Jul 15;9:1481. doi: 10.1038/s42003-026-10653-7 (PMC13740232; doi:10.1038/s42003-026-10653-7)
Supplement: Supplementary file 3 [file 42003_2026_10653_MOESM3_ESM.pdf]

nature portfolio

Corresponding author(s): Steven Prescott

Last updated by author(s): 2026/05/05

Reporting Summary

Nature Portfolio wishes to improve the reproducibility of the work that we publish. This form provides structure for consistency and transparency in reporting. For further information on Nature Portfolio policies, see our Editorial Policies and the Editorial Policy Checklist.

Statistics

For all statistical analyses, confirm that the following items are present in the figure legend, table legend, main text, or Methods section.

n/a	Confirmed	
☐	☒	The exact sample size (n) for each experimental group/condition, given as a discrete number and unit of measurement
☐	☒	A statement on whether measurements were taken from distinct samples or whether the same sample was measured repeatedly
☒	☐	The statistical test(s) used AND whether they are one- or two-sided<br>*Only common tests should be described solely by name; describe more complex techniques in the Methods section.*
☒	☐	A description of all covariates tested
☒	☐	A description of any assumptions or corrections, such as tests of normality and adjustment for multiple comparisons
☐	☒	A full description of the statistical parameters including central tendency (e.g. means) or other basic estimates (e.g. regression coefficient) AND variation (e.g. standard deviation) or associated estimates of uncertainty (e.g. confidence intervals)
☒	☐	For null hypothesis testing, the test statistic (e.g. F, t, r) with confidence intervals, effect sizes, degrees of freedom and P value noted<br>*Give P values as exact values whenever suitable.*
☒	☐	For Bayesian analysis, information on the choice of priors and Markov chain Monte Carlo settings
☒	☐	For hierarchical and complex designs, identification of the appropriate level for tests and full reporting of outcomes
☒	☐	Estimates of effect sizes (e.g. Cohen's d, Pearson's r), indicating how they were calculated

Our web collection on statistics for biologists contains articles on many of the points above.

Software and code

Policy information about availability of computer code

Data collection	OmniPlex Data Acquisition System (Plexon)
Data analysis	Offline Sorter v5 Software (Plexon), MATLAB (MathWorks, R2022), Python 3.10

For manuscripts utilizing custom algorithms or software that are central to the research but not yet described in published literature, software must be made available to editors and reviewers. We strongly encourage code deposition in a community repository (e.g. GitHub). See the Nature Portfolio guidelines for submitting code & software for further information.

Data

Policy information about availability of data

All manuscripts must include a data availability statement. This statement should provide the following information, where applicable:

- Accession codes, unique identifiers, or web links for publicly available datasets
- A description of any restrictions on data availability
- For clinical datasets or third party data, please ensure that the statement adheres to our policy

The original dataset (i.e. spike times) generated and analysed in the current study are available from the corresponding authors on reasonable request.

Research involving human participants, their data, or biological material

Policy information about studies with human participants or human data. See also policy information about sex, gender (identity/presentation), and sexual orientation and race, ethnicity and racism.

Reporting on sex and gender	All experiments were conducted in male rats, as stated at the start of the Methods. We are not aware of any sex differences in coding by primary afferents, and have not identified any sex differences in our previous work (Medlock et al. J Neurosci 2024).
Reporting on race, ethnicity, or other socially relevant groupings	N/A
Population characteristics	N/A
Recruitment	N/A
Ethics oversight	All procedures were approved by the Animal Care Committee of the Hospital for Sick Children (protocol #47072)

Note that full information on the approval of the study protocol must also be provided in the manuscript.

Field-specific reporting

Please select the one below that is the best fit for your research. If you are not sure, read the appropriate sections before making your selection.

☒ Life sciences ☐ Behavioural & social sciences ☐ Ecological, evolutionary & environmental sciences

For a reference copy of the document with all sections, see nature.com/documents/nr-reporting-summary-flat.pdf

Life sciences study design

All studies must disclose on these points even when the disclosure is negative.

Sample size	The number of recorded neurons (26) is reported. Sample size is not important in the typical sense; instead, we explain how data from different neurons are used to create virtual ensembles, from which we test discrimination.
Data exclusions	Data are reported for all units displaying cleanly sorted spikes and for which we completed testing of >50% of all stimulus intensity-frequency combinations.
Replication	In the context of this study, we tested that different decoders yielded similar results, or that ISI histograms generated in different ways yielded similar results. In other words, variations in the analysis methods were thoroughly explored to ensure results were robust.
Randomization	N/A. Animals were not allocated to different groups for comparison.
Blinding	N/A. Animals were not allocated to different groups for comparison.

Reporting for specific materials, systems and methods

We require information from authors about some types of materials, experimental systems and methods used in many studies. Here, indicate whether each material, system or method listed is relevant to your study. If you are not sure if a list item applies to your research, read the appropriate section before selecting a response.

Materials & experimental systems

n/a	Involved in the study	
☒	☐	Antibodies
☒	☐	Eukaryotic cell lines
☒	☐	Palaeontology and archaeology
☐	☒	Animals and other organisms
☒	☐	Clinical data
☒	☐	Dual use research of concern
☒	☐	Plants

Methods

n/a	Involved in the study	
☒	☐	ChIP-seq
☒	☐	Flow cytometry
☒	☐	MRI-based neuroimaging

Animals and other research organisms

Policy information about studies involving animals; ARRIVE guidelines recommended for reporting animal research, and Sex and Gender in Research

Laboratory animals	Adult (200-350 g) male Sprague-Dawley rats were obtained from Charles River, Montreal.
Wild animals	N/A
Reporting on sex	All experiments were conducted in male rats. We are not aware of any sex differences in coding by primary afferents, and have not identified any sex differences in our previous work (Medlock et al. J Neurosci 2024).
Field-collected samples	N/A
Ethics oversight	All procedures were approved by the Animal Care Committee of the Hospital for Sick Children (protocol #47072)

Note that full information on the approval of the study protocol must also be provided in the manuscript.

Plants

Seed stocks	N/A
Novel plant genotypes	N/A
Authentication	N/A
